# Hydrogels with Essential Oils: Recent Advances in Designs and Applications

**DOI:** 10.3390/gels10100636

**Published:** 2024-09-30

**Authors:** Mariana Chelu

**Affiliations:** “Ilie Murgulescu” Institute of Physical Chemistry, 202 Spl. Independentei, 060021 Bucharest, Romania; mchelu@icf.ro

**Keywords:** hydrogels, essential oils, encapsulation, biomedical applications, cosmetics, dentistry, active food packaging, restoration of cultural heritage

## Abstract

The innovative fusion of essential oils with hydrogel engineering offers an optimistic perspective for the design and development of next-generation materials incorporating natural bioactive compounds. This review provides a comprehensive overview of the latest advances in the use of hydrogels containing essential oils for biomedical, dental, cosmetic, food, food packaging, and restoration of cultural heritage applications. Polymeric sources, methods of obtaining, cross-linking techniques, and functional properties of hydrogels are discussed. The unique characteristics of polymer hydrogels containing bioactive agents are highlighted. These include biocompatibility, nontoxicity, effective antibacterial activity, control of the sustained and prolonged release of active substances, optimal porosity, and outstanding cytocompatibility. Additionally, the specific characteristics and distinctive properties of essential oils are explored, along with their extraction and encapsulation methods. The advantages and disadvantages of these methods are also discussed. We have considered limitations due to volatility, solubility, environmental factors, and stability. The importance of loading essential oils in hydrogels, their stability, and biological activity is analyzed. This review highlights through an in-depth analysis, the recent innovations, challenges, and future prospects of hydrogels encapsulated with essential oils and their potential for multiple applications including biomedicine, dentistry, cosmetics, food, food packaging, and cultural heritage conservation.

## 1. Introduction

Hydrogels form a unique category, being among the most modern multifunctional materials that can be applied in numerous fields. As a result, they have captured the interest of many scientists in various research fields. Hydrogels are essentially 3D cross-linked networks formed of hydrophilic polymeric materials that can retain large volumes of water and fluids [1]. They can be formulated from both synthetic polymers and biopolymers. Hydrogels based on natural biodegradable polymers, such as polysaccharides, polypeptides, and proteins have many advantages over synthetic ones and have gained particular importance lately [2]. One of the advantages is the porous macromolecular structure, which can be easily adjusted so that the hydrogels can incorporate different bioactive compounds and then release them in a controlled manner.

Due to their special properties, the so-called intelligent hydrogels have the ability to swell in an aqueous environment, show sensitivity to temperature, light, pH variations and other stimuli, self-healing, and shape memory [3].

Through different strategies, such as molecular design, cross-linking techniques, or the incorporation of different bioactive compounds, the properties and functions of hydrogels can be adapted for a wide variety of applications. They are widely used in the biomedical field for wound healing and tissue engineering, as well as drug delivery systems, pharmaceutical products, the food industry, cosmetics, hygiene products, and dentistry, etc.

Similarly to the remarkable class of hydrogels, essential oils (EOs) have shown an extraordinary increase in scientific interest in recent years, providing huge potential for a diverse range of modern applications, including cutting-edge fields such as nanotechnology, bioengineering, and biomedicine. Extracted from aromatic plants, EOs are hydrophobic products of high concentration and contain molecules with low molecular weight. The extraordinary therapeutic potential of EOs is due to the biological activity of their volatile chemical components (terpenoids, terpenes, and other aromatic compounds) and non-volatile (hydrocarbons, fatty acids, sterols, carotenoids, waxes, and flavonoids) [4].

This review brings a new perspective, scrutinizing the potential to incorporate different essential oils into hydrogels in order to develop efficient delivery systems for bioactive molecules of natural origin. Through this versatile delivery method, new products with improved bioactive activity can find use for many types of applications to provide both cost-effectiveness and high efficiency.

At present, the global production of essential oils is driven by the strong demand for consumption, from natural options to synthetic antioxidants, due to remarkable biological properties, including antimicrobial, antioxidant, anti-inflammatory, antiviral, and antitumor effects [5]. These distinctive qualities pave the way for promising opportunities in utilizing EOs across different fields.

As potent curative agents, EOs offer a viable alternative to synthetic drugs, particularly for their antimicrobial effectiveness against a wide range of pathogenic microorganisms. They are most commonly applied in the biomedical and pharmaceutical sectors.

Since ancient times, the sensory and pharmacological activities of essential oils have been studied for their use in preventive and curative treatments, particularly in cosmetics and countless personal care products.

In the food industry, EOs are used on a large scale, as a beneficial mechanism to combat undesirable microorganisms in food products. The natural bioactive capacities of EOs, such as antimicrobial and antioxidant properties, together with their aroma, flavor, or spicy taste, make them suitable for use as preservatives in the food industry.

EOs can be incorporated into different food systems to increase the shelf life of food while maintaining its quality. They can also be encapsulated in the form of edible coatings or films, to mitigate microbial development on the surface and protect the environment from synthetic packaging.

However, EOs cannot be applied directly, in their raw form, because they are unstable volatile compounds, fragile, and of very high concentration. They require special post-obtaining conditions so that the original chemical profile is not modified by different environmental conditions (light, heat, oxidation) [6].

Consequently, EOs can be encapsulated in different hydrogel matrices to prolong their effective biological activity and ensure their release in a controlled and sustained manner.

Hydrogels are versatile platforms with an adjustable structure, of a non-toxic nature that can be used safely [7]. The high-water content, the sustainable nature of the constituent polymers, and the ability to incorporate EOs give hydrogels favorable biocompatibility and key structural characteristics, making them suitable for a wide range of implementation [8].

This review aims to showcase significant research and findings through a detailed analysis of the main applications of essential oil-embedded hydrogels. Therefore, this review article provides a comprehensive overview of recent studies on obtaining hydrogels, and EOs, as well as their incorporation into hydrogel matrices, for a multitude of uses. These include fields such as biomedicine, dentistry, cosmetics, functional food products, food packaging, and even the preservation of stone cultural heritage.

## 2. Preparation of Hydrogels

Hydrogels are systems made up of polymers and solvents obtained in the form of 3D cross-linked networks. Cross-linking can be formed (i) physically, (ii) chemically or (iii) by ionizing radiation [9].

The main advantage of hydrogels obtained by the physical method is the absence of crosslinking agents which eliminates the risk of possible residual toxicity [9,10].

However, a notable disadvantage is that physically cross-linked hydrogels are reversible. Between the polymer chains, there are temporary bonds that appear in response to composition, pH, or temperature changes. Moreover, these hydrogels show weak mechanical and viscoelastic properties. Depending on the size of the polymer particles and the nature of the solvent in the 3D network, there may be hydrogen bonds, van der Walls bonds, electrostatic, hydrophobic interactions, or between-polymer chains with local crystallite generation [9].

Chemically obtained hydrogels are created by covalent cross-linking between the existing polymer chains. This leads to a stable or irreversible bond. Chemical cross-linking can be produced using various methods, including addition polymerization, photopolymerization, volume condensation, plasma or electromagnetic radiation, or interpenetration [10].

In general, a cross-linking agent with a multifunctional role is used, which unites the monomeric units and leads to the development of the polymer chain [10]. Chemically cross-linked hydrogels are advantageous due to their resistance to degradation and improved mechanical and viscoelastic properties compared to physically cross-linked ones [9]. On the other hand, hydrogels, obtained by anionic or cationic polymerization have as their main disadvantage the sensitivity to water, respectively, the limitation to non-polar monomers [9].

In the case of hydrogels made by radiation, they can be obtained at ambient temperature and physiological pH, even without a crosslinking agent, which makes them suitable to be applied in a wide range of biomedical, food, or cosmetic applications [11].

**Figure 1:**
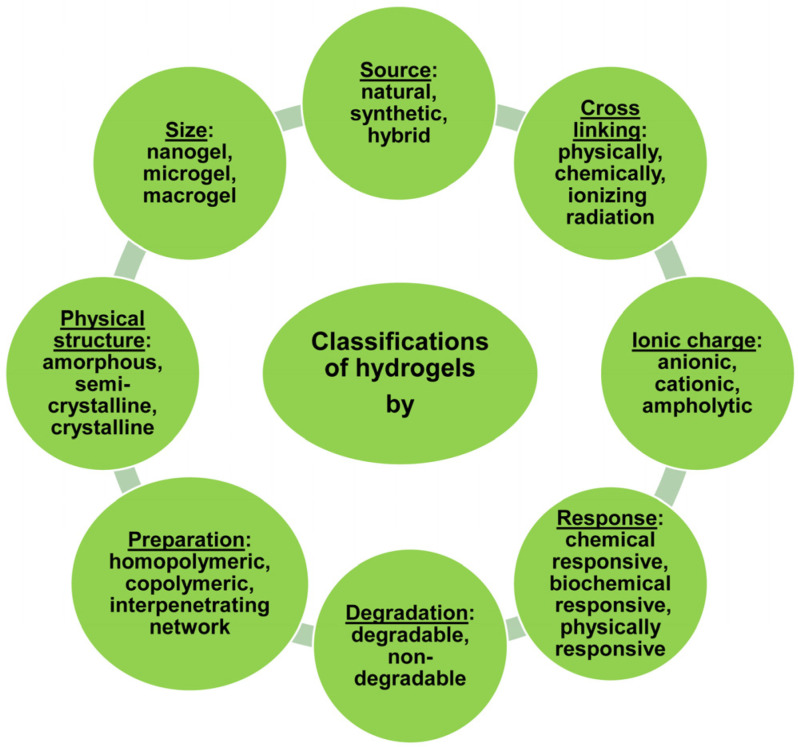
Classifications of hydrogels.

Hydrogels can be categorized according to various characteristics, providing deeper insight into their properties and potential uses (Figure 1). These characteristics include [9]:

➢ According to source: natural, synthetic, or hybrid.
➢ According to polymer structure: linear, branched, or cross-linked.
➢ According to physical appearance: macroporous, microporous, or nanoporous.
➢ According to charge: neutral, anionic (negatively charged), cationic (positively charged), or amphoteric, meaning they contain both positive and negative charges.
➢ According to responsiveness to stimuli: Hydrogels can also be classified by their sensitivity to external stimuli such as temperature, pH, light, or electric fields. Called “smart hydrogels,” these materials can undergo reversible changes in their structure or properties when subjected to these environmental influences.
➢ According to water content: superabsorbent hydrogels, and less moisture.
➢ According to degradability: biodegradable, and non-biodegradable.

Polymers play an important role in the matrix of hydrogels, greatly influencing their properties. They can be divided into two main categories: natural polymers and synthetic polymers. Biodegradable polymeric materials, both natural and synthetic, have received more interest lately, due to the importance of environmentally friendly products and the possibility of their application in many fields such as biomedicine, pharmaceuticals, or agriculture [12].

The creation of advanced materials formulated from bioavailable and renewable raw materials, including waste, has been increasingly promoted, in agreement with the 12 principles of Green Chemistry and the achievement of the Sustainable Development Goals provided for the UN 2030 Agenda.

Natural polymers are polymer molecules of biological origin that can be obtained from different sources such as animals, bacteria, microorganisms (algae and fungi), or plants. The chemical structures of natural polymers are composed of monomers of amino acids, nucleotides, esters, or monosaccharides, that are covalently coupled to form peptides, polyphenols, polyesters, or polysaccharides [13].

Because they are similar to the components of the extracellular matrix (ECM), natural polymers have a reduced toxicity, with a low risk of causing adverse reactions. This biocompatibility has determined their widespread use in numerous biomedical, pharmaceutical, and cosmetic applications, as additives in textile products, and in food or agriculture [14].

The most used natural polymers include sodium alginate, starch, gelatin, chitosan, collagen, hyaluronic acid, κ-carrageenan, cellulose, gum arabic, silk, fibrin, and bacterial polyesters [15,16].

Synthetic polymers are created artificially in laboratories and can be mainly classified as thermoplastic and thermosetting polymers and elastomers. They are very often found in multiple fields, such as packaging and construction, as plastic materials, fibers, elastomers, or adhesives. As synthetic polymers, we can mention polyvinyl alcohol, poly(lactic acid), polyvinylpyrrolidone, poly(ε-caprolactone), polyurethane, polyethylene glycol, polyethylene oxide, poly(L-lactide-co-caprolactone), carboxymethyl cellulose and poly(vinylidene fluoride) [17]. Among them, some synthetic polymers are biocompatible and biodegradable, such as poly(lactic acid), carboxymethyl cellulose, poly(acrylic acid), poly(vinyl alcohol), or polyethylene glycol. Some of them have shown antitumor, antibiotic, antiviral, or antithrombotic activities and are often used as drug carriers, implants, diagnostic imaging agents, or as bio-ink in 3D printing for various biological scaffolds [18,19].

A new approach in hydrogel engineering is the design of complex systems through which hybrid hydrogels are obtained that incorporate both natural and synthetic polymers, but also other functional components [20]. The hybrid hydrogels that have been developed are capable of integrating nano- or microstructures, allowing for targeted action, controlled transport, and an adjustable release profile.

Hybrid nanogels have the ability to respond faster than macroscopic ones to environmental variations, proving their usefulness especially in biomedical applications such as therapies for tissue engineering, transport and delivery of chemicals in cancer therapy, or in optical detection [21].

## 3. Methods of Obtaining Essential Oils

Since ancient times, people have used EOs because they were believed to contain essential components that are necessary for healing and prolonging life. Alchemists referred to them as the “quintessence of plants”.

Essential oils are aromatic oil liquids in the form of complex natural mixtures of different polar and non-polar compounds, made from natural raw material of plant origin. The main chemical constituents of essential oils are volatile, lipophilic, and odoriferous substances that are commonly found in different parts of plants (leaves, flowers, fruits, or stems), giving them specific properties [22]. Since the beginning, aromatic plants have been used empirically as spices in kitchens, in perfumes, cosmetics, and aromatherapy, for preventive, curative, or therapeutic purposes. With the advent of distillation centers, the methods of obtaining essential oils have advanced and the therapeutic benefits of EO have been scientifically evaluated [23].

The different production techniques involve (i) hot distillation, with water or steam, (ii) cold or dry distillation, and (iii) through various mechanical processes. While the yield of obtaining pure essential oils from aromatic plants is very low, their price is commensurate with their natural bioactivity and implicit pharmaceutical and therapeutic benefits.

Over time, it has been proven that essential oils contain over 300 different aromatic components, showing an extremely varied therapeutic potential [24]. In practice, these capabilities of essential oils have been continuously explored and harnessed on a large scale for a wide range of multiple applications that have been successfully integrated into a variety of industries and contexts. The chemical composition of essential oils can vary, even for the same species, depending on certain parameters such as climatic factors, soil characteristics, harvesting conditions, post-harvest treatment, or the extraction methods used [24].

The uniqueness and importance of EOs are given by their medicinal and bioactive compounds, as well as by the other valuable constituents. EOs show significant variability in their composition, both in terms of quality and quantity. Since this variability is heavily influenced including on the extraction method used, it is crucial to identify optimal, and especially non-toxic extraction techniques.

This chapter will offer a concise summary of the main extraction methods, including both traditional and modern approaches, which are continuously being refined for improvement.

The main compounds discovered in EOs are terpenoid derivatives (80%) and phenylpropanoids (which give the specific spicy smell and aroma) [25].

There are several ways by which EOs are extracted from aromatic plants. The selection of an extraction method is influenced by the plant’s texture and characteristics, the specific essential oils being targeted, and the intended application of the final product. Every method demonstrates its own advantages and drawbacks.

Some classical EO extraction methods, also called conventional, have been practiced for hundreds of years and include steam distillation, water distillation, combined water and steam distillation, cohobation (or repeated distillation), maceration, cold pressing, and enfleurage. The limitations of these methods are mainly represented by low extraction yields, thermal degradation, or the need to use high mechanical power [23].

Other more recent methods, also called alternatives, try to demonstrate their efficiency in operation, to be ecological and viable from an economic point of view. Among these can be listed extraction with solvents, supercritical CO_2_, or resins, and fractional distillation, percolation, and the phytonic process (uses a new solvent based on hydrofluorocarbon 134) [23]. The main benefits of supercritical extraction with CO_2_ are its low cost and non-corrosive nature, which enables the production of thermally unstable EOs at an industrial level with a high yield. In addition, it is an ecological way by which safe EOs can be produced to be used in various applications in the food industry.

New extraction methods use “green concepts” to extract valuable components from aromatic plants. They act in accordance with the United Nations 2030 strategy, pursuing sustainable developments by reducing waste, using discarded by-products, recycling them, and reducing the carbon footprint in processing, which will have a positive impact towards a cleaner environment. The global EO market has been continuously growing, reaching USD 7.51 billion in 2018, and is expected to grow at a CAGR of over 9% between 2019 and 2026 [26].

Innovative techniques that respect these green concepts include ultrasound-assisted extraction of bioactive compounds, microwave-assisted extraction of essential oils, high-pressure liquid extraction, sub- and supercritical fluid extraction, pulsed electric fields, and high-voltage electric discharges [27].

Essential oils are categorized into three main categories which include (i) terpenes, (ii) terpenoids, and (iii) phenylpropanoids in their chemical composition. Terpene constituents can be classified into two primary groups: (i) components that have a hydrocarbon structure (such as monoterpenes diterpenes and sesquiterpenes) and (ii) those that are oxygenated, including acids, aldehydes, alcohols, esters, ketones, lactones, oxides, and phenols [28]. Among the most common terpenes, we can distinguish limonene, sabinene, α-pinene and p-cymene, for example in thyme and oregano, but also in lemon, grapefruit, eucalyptus, and rosemary EOs. Carvacrol, citronellal, carvone, and thymol are all terpenoids that are present in EOs like mint, lavender, tea tree, chamomile, or geranium. Clove, jasmine, rose, or pepper EOs contain phenylpropanoids that can be identified as cinnamaldehyde, eugenol, safrole, and vanillin. EOs also includes other components derived from amino acids, such as alanine, leucine, isoleucine, methionine, and valine [25].

The primary drawbacks of using EOs include their volatility, high sensitivity, poor stability, high sensitivity, and vulnerability to degradation at processing temperatures. To address these challenges, the encapsulation of EO in polymeric matrices improves their bioactivity, stability, and water solubility, and enables long-term sustained delivery across various applications [29].

## 4. Encapsulation of Essential Oils in Hydrogels

Most essential oils cannot be applied through direct contact with biological systems, because they can be irritating or even toxic in certain cases. Incorporating essential oils directly into hydrophilic matrices is not beneficial due to their hydrophobic nature, which diminishes the inherent bioactivity of their components, leading to the use of high concentrations to be functional. To preserve their biological activity over an extended period, particularly for biomedical applications, EOs should be encapsulated in various systems, such as lipidic nanoparticles, liposomes, films, emulsion gels, oil-in-water emulsions, or spray-dried microparticles [30].

Through encapsulation, risks due to possible toxicity are reduced, ensuring a safe delivery system. Moreover, this method increases the biological activities and efficiency of particularly volatile EOs through better absorption. In addition, encapsulation has often been widely used as a way of protecting essential oils, prolonging their active biocapacity and efficient delivery, which offers the possibility of their implementation in the medical, pharmaceutical, cosmetic, and food fields [31].

These benefits can be achieved by employing different techniques to encapsulate es- essential oils with diverse bioactivities [26,32]: (i) chemical; (ii) physico-chemical; (iii) mechanical; (iv) ultrasound-assisted emulsification; and (v) electrostatic extrusion.

An example is thyme EO, which is often used due to its therapeutic properties. Thyme (*Thymus vulgaris* L.) is a plant native to the Mediterranean area with both dietary and medicinal uses. It contains many polyphenolic compounds of biological interest, such as carvacrol, 5-isopropyl-2-methylphenol, and a p-cymene derivative with a characteristic smell, with antioxidant, antimicrobial, antidiabetic, anti-inflammatory, immunomodulatory, and anticancer bioactivities [33,34]. Thyme EO was encapsulated in the first step in sodium caseinate nanomicelles by a physical method [35]. Then, in the second step, these nanomicelles were introduced into the preparation of a hydrogel. This formulation aimed to improve the stability and protect the bioactivity of thyme EO. Finally, a gelatin nanocomposite hydrogel as a drug delivery platform was obtained, having antibacterial potential for wound healing both in vitro and in vivo [35].

The ionic gelation method, achieved by ionic bonding between alginate and some divalent cations, led to the creation of a biocompatible hydrogel material in the form of alginate microspheres that encapsulated thyme and calendula EO [36]. The study investigated the loading capacity, the encapsulation efficiency of EO, and the dissolution of microspheres under simulated digestion conditions.

Lipid matrices offer a suitable and stable environment for incorporating EOs and ensuring their controlled release [37]. In particular, solid lipid nanoparticles have aroused special interest for the encapsulation of bioactive compounds due to their large surface area and the potential to facilitate the protection of bioactive constituents in ambient conditions.

An interesting method for obtaining lipid matrices is homogenization at high shear followed by the ultrasonication method [37]. Thus, chitosan and polyvinyl alcohol hydrogels containing solid lipid nanoparticles loaded with EOs of *Origanum vulgare* and *Thymus vulgaris* were formulated and investigated as alternatives to synthetic fungicides. The materials made with EOs content have demonstrated abilities to reduce the infestation with phytopathogenic fungi responsible for the degradation of perishable fruits [37].

A new work investigated the capabilities of *Perilla frutescens* (L.), the annual aromatic plant cultivated and used for thousands of years in traditional medicine or as food. In the first step, microcapsule powders of *Perilla frutescens* (L.) [38] EOs were prepared by the spray drying method of a wall material (octenyl succinic anhydride starch). In the next step, they were further encapsulated with sodium alginate and chitosan by the polyelectrolyte complex coacervate method, obtaining stable hydrogel balls for aqueous and acidic food formulations with a complete and prolonged release of the encapsulated EOs [38].

Hydrophobic clove EO was loaded in situ into a hydrophilic chitosan polymer matrix to obtain functional coatings as food packaging [32]. By using this method, bioactive materials were obtained without the need for crosslinking agents.

The electrostatic extrusion technique that was applied to encapsulate fennel EO in an alginate polymer matrix, together with the incorporation of a whey protein followed by freeze-drying, is an original approach to improve the encapsulation efficiency and loading capacity [39]. The encapsulated EO maintained its qualitative appearance by keeping 58.95% of the volatile compounds [39].

## 5. Applications of Hydrogel Materials Enriched with Essential Oil

The exploration and application of the bioactivities of essential oils as natural phytotherapeutic agents in various biomedical fields arose from the need to develop alternative therapeutic approaches to traditional synthetic treatments.

Hydrogels are ideal host matrices for some limitations of EOs, such as volatility, high sensitivity to environmental factors, and lower stability [26]. Together, EOs and hydrogels are biocompatible and biodegradable materials, which demonstrate remarkable physicochemical properties and antibacterial, antioxidant, anti-inflammatory, and anti-cancer activities [40]. The porous 3D structure of hydrogels facilitates the incorporation of essential oils through hydrophobic interactions, enabling their sustained and controlled release in response to various stimuli such as hydrolytic and enzymatic activity, pH changes, or temperature variations [41].

The beneficial combination of essential oils with the engineering of hydrogels can be an advanced approach to the design and development of the next generation of hybrid biomedical systems that embed natural therapeutic compounds.

### 5.1. Biomedical Applications

#### 5.1.1. Topical or Transdermal Delivery Systems

Natural polysaccharides are among the most widely used biopolymers in biomedical applications due to their biocompatibility, bioactivity, biodegradability, and exceptional rheological and biomucoadhesive properties. These attributes make them ideal for developing a wide variety of topical formulations, for wound healing, or as effective and inexpensive drug delivery systems [42]. Additionally, marine polysaccharides enhance hydrogel formation capabilities, making them particularly effective for skin applications in treating various dermatological conditions.

**Figure 2:**
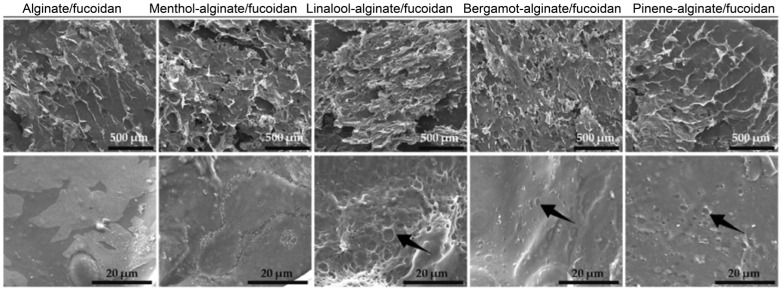
SEM images of the hydrogel samples [43].

Alginate and fucoidan hydrogels loaded with menthol, L-linalool, bergamot oil, and β-pinene essential oils have been developed to improve skin permeability [43]. The aim of the study was to evaluate the way in which these EOs influence the penetration of the active ingredients through the skin, and the effect of the composition, in order to create effective formulations for topical or transdermal administration [43]. The porous morphology of the prepared hydrogels, presented in Figure 2, could be due to the lyophilized oil droplets, which can lead to these structures. Menthol, a cyclic monoterpene, is widely recognized for its ability to improve skin permeability by disrupting intercellular lipids in the stratum corneum. Bergamot EO (*Citrus bergamia*) is mainly composed of limonene, linalyl acetate, and linalool with anti-inflammatory properties and b-pinene, a bicyclic monoterpene, with antioxidant, anti-inflammatory, and analgesic effects. Combining the activities of EOs like menthol, L-linalool, bergamot oil, and β-pinene can indeed be a powerful strategy for overcoming the skin barrier and treating inflammation. Each of these essential oils has unique properties that, when combined, can work synergistically to enhance skin permeability and provide anti-inflammatory benefits [43].

#### 5.1.2. Antimicrobial and Anti-Inflammatory Activity

Oral candidiasis is a fungal infection primarily produced by *Candida* species for which there is a rather limited antifungal treatment. This condition is particularly challenging to manage due to the limited availability of effective antifungal treatments and the potential for these treatments to cause adverse effects and contribute to the development of antifungal resistance.

Encapsulating biocides within hydrogels is an effective strategy for targeted delivery, offering controlled release and enhanced therapeutic effects. Specifically, using methylcellulose-based hydrogels incorporated with *Melissa officinalis* EO can enhance antimicrobial efficacy while maintaining biocompatibility with biological tissues [44]. The hydrogel formulation based on methylcellulose with *Melissa officinalis* EO demonstrated both antimicrobial activity and antifungal potential, making it an effective treatment for inhibiting oral candidiasis [44].

**Figure 3:**
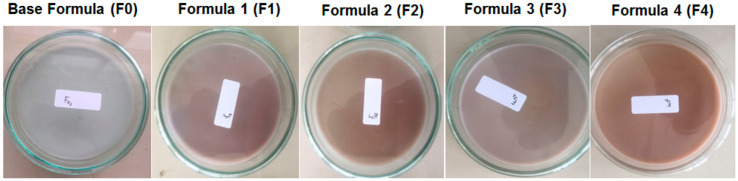
Photo of hydrogel films based on polyvinyl alcohol–cornstarch–patchouli oil and Ag nanoparticles [45].

A complex study focused on the development of hydrogel films made from a combination of polyvinyl alcohol (PVA), corn starch, patchouli oil, and silver nanoparticles (Figure 3) [45]. These materials were chosen for their bioactive properties, particularly their effectiveness against *Staphylococcus aureus* and *Staphylococcus epidermidis*, both of which are common bacteria responsible for various infections, including skin and soft tissue infections. The nanoparticles were prepared by green synthesis, in the presence of both aqueous and methanolic extracts from patchouli plants (*Pogostemon cablin* Benth). The use of cross-linked polymeric hydrogel films with glutaraldehyde and containing biosynthesized silver nanoparticles with phytochemicals presents an advanced approach to developing antimicrobial materials [45].

**Figure 4:**
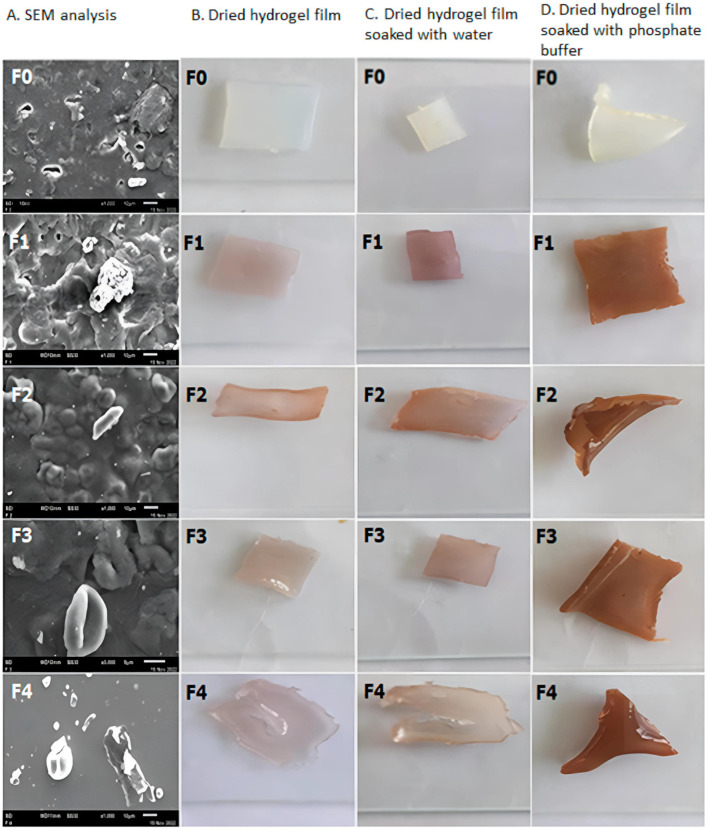
SEM images of hydrogel films based on polyvinyl alcohol/corn starch/patchouli oil (**A**) with Ag nanoparticles (samples F0–F4); (**B**) Dry samples; (**C**) Dry samples soaked in water; (**D**) Dry samples soaked with phosphate buffer [45].

Figure 4 shows SEM and photo images during the swelling experiments.

Acne Vulgaris is a common inflammatory skin condition that affects many young individuals and often persists into adulthood. Traditional acne treatments, which mainly rely on antibiotics, have shown limited effectiveness and frequently disrupt the balance of the skin microbiome. Recent research suggests that essential oils and herbs could offer promising benefits for treating acne, a long-lasting inflammatory condition that can lead to scarring [46].

Thyme EO has excellent antibacterial and antioxidant properties that are suitable for inflammatory skin conditions such as acne [47]. Obtained by steam distillation of the flowering stems, Thyme EO contains thymol (37–55%) and 0.5-carvacrol (0.5–5.5%). These biocomponents have antibacterial activity, easily penetrating the lipid layer. Although Thyme EO is recognized for its insolubility in water, high volatility, and tendency to degrade rapidly when exposed to air, light, or high temperatures for long periods, the extraordinary potential of this plant has been explored in numerous studies.

**Figure 5:**
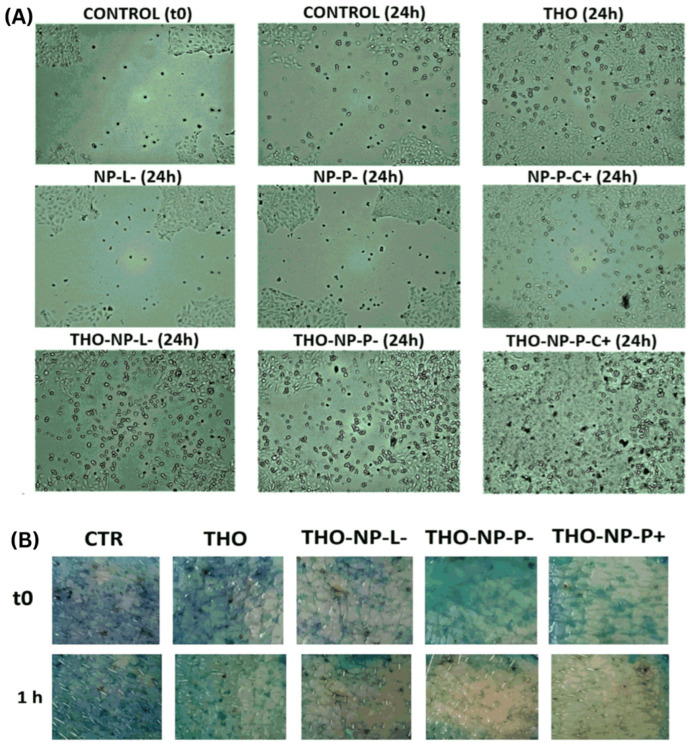
Wound healing activity: (**A**) for in vitro scratch assay in HaCaT cell lines. Images were taken before (Control t0) and 24 h after incubation: untreated samples (control and 24 h) and treated samples, respectively thyme oil (THO) and functionalized hydrogels and their corresponding empty NPs. (**B**) Skin surface showed a reduction in methylene blue following ex vivo antioxidant activity of the samples [47].

In a recent research, Thyme EO was encapsulated in biodegradable nanoparticles of poly-(D,L)-(lactic-co-glycolic acid) for skin and pharmaceutical applications [47]. Through functionalization, the nanosystems remained stable for a period of 6 months, by cold storage. In vitro, ex vivo, and in vivo evaluations on human volunteers indicated that Thyme EO demonstrated excellent antioxidant activity and healing of skin inflammation without leaving acne scars (Figure 5A,B) [47].

#### 5.1.3. Wound Dressing Applications

Numerous formulations of composite hydrogels have been studied as intricate systems composed of biopolymers, incorporating various bioactive elements from essential oils. These platforms, exhibiting synergistic properties, are being explored for use as advanced wound dressings with enhanced therapeutic potential [48].

**Figure 6:**
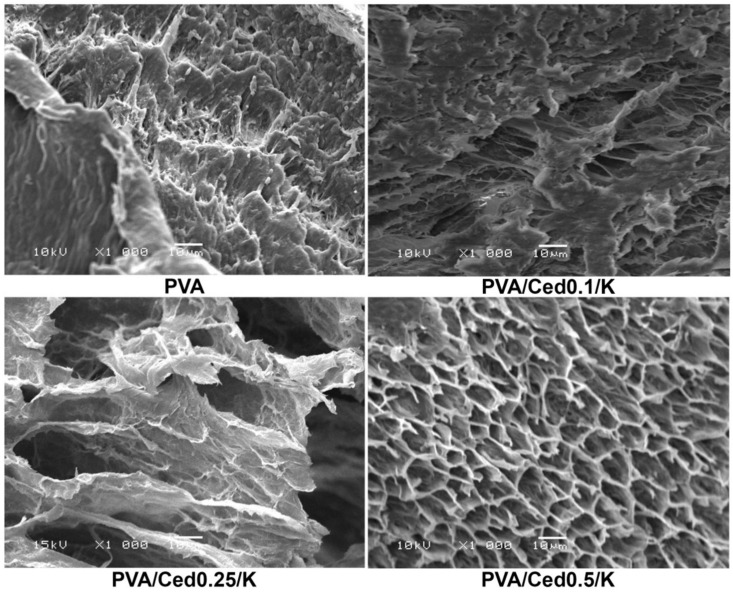
SEM micrographs at 1000× magnifications showing the microstructure in cross-sectional of composite sponges [49].

Cedarwood EO obtained from several types of conifers (e.g., *Cedrus* sp. and *Juniperus* sp.) is a mixture of safe organic chemicals with pesticidal and preservative properties. In order to develop effective hemostatic and antibacterial dressings for treating wounds, composite porous sponges were designed [49]. Polyvinyl alcohol was physically cross-linked with kaolin and incorporated cedar essential oil, through a freeze–thaw approach, yielding sponge hydrogels with distinct lamellar architectures. The addition of cedar and kaolin in the formulation improved the pore sizes and structure of the resulting sponges (Figure 6). Studies have shown the biocompatibility of these sponges, improved antibacterial activity against *Bacillus cereus* and *Escherichia coli*, and high free radical scavenging capacity and hemostatic performance [49].

Clove (*Syzygium aromaticum* L. Myrtaceae) EO possesses significant biological activities beneficial to human health, such as antimicrobial, antioxidant, and insecticidal properties. Consequently, it has attracted considerable attention for its widespread use in the medical world, perfume, cosmetic, flavoring, and food industries [50]. It can be extracted by (i) hydrodistillation, (ii) steam distillation, (iii) ultrasound-assisted extraction, (iv) microwave-assisted extraction, (v) cold pressing, or (vi) supercritical fluid extraction. The extraction methods used determine the concentration of primary volatile compounds in clove essential oil and organic clove extracts. It contains mostly eugenol (at least 50%), respectively, eugenyl acetate, β-caryophyllene, and α-humulene (10–40%).

**Figure 7:**
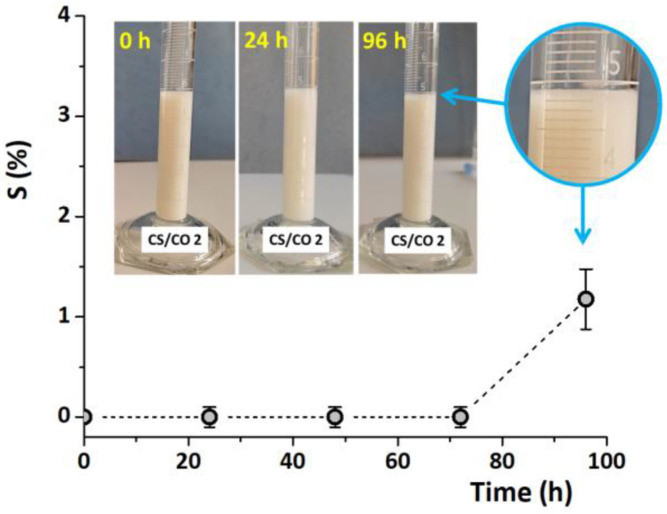
Photos of the stable chitosan emulsion with clove EO content (after 0–4 days) kept in the dark and ambient conditions [52].

**Figure 8:**
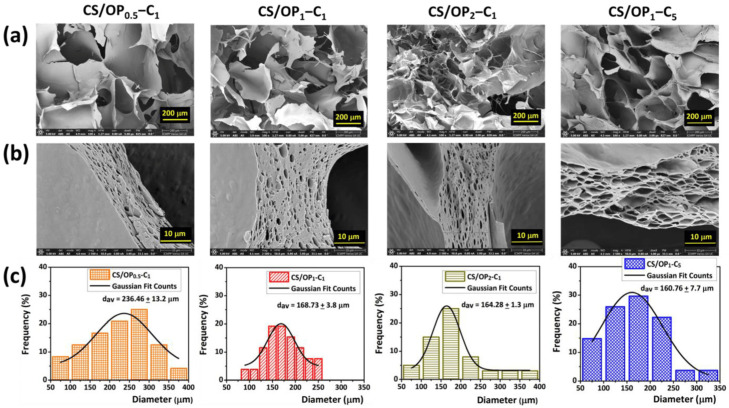
Cross-sectional SEM images of chitosan and oxidized pullulan-based hydrogels loaded with clove EO: overview (**a**), wall detail (**b**), and pore size distribution with corresponding diagrams (**c**) [52].

The development of different materials for biomedical applications has been in continuous growth lately [51]. Thus, the biological capabilities of clove EO were used in a very interesting recent study for the generation of hydrogels as wound dressings. The hydrogels were loaded with cloves EO by combining covalent and physical cross-linking methods. In the first step, EO was emulsified and stabilized in a chitosan-based solution, which was further strengthened by covalent cross-linking of the Schiff base with another polysaccharide, namely oxidized pullulan (Figure 7). In the next step, several freeze–thaw cycles were performed to stabilize the cloves EO in the physically cross-linked polymer walls. The hydrogels formed with a sponge-like porous structure (Figure 8) exhibited outstanding elasticity [52].

**Figure 9:**
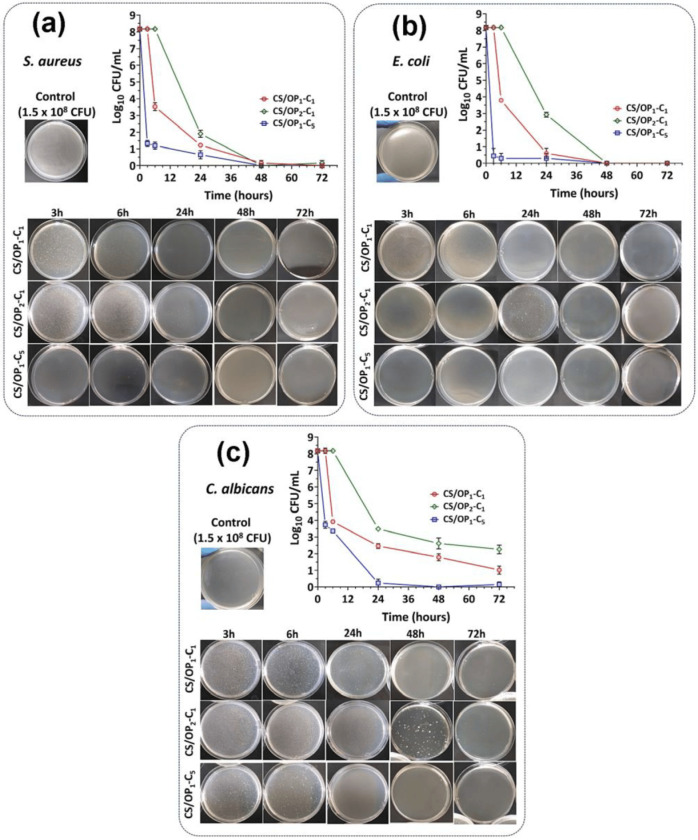
Antibacterial effect of clove oil-loaded hydrogels evaluated by the time-kill method, after 3–72 h of incubation with *S. aureus* (**a**), *E. coli* (**b**), and *C. albicans* (**c**) [52].

The antibacterial activity of hydrogels containing clove essential oil was evaluated by the time-kill method, for different incubation time intervals, against three bacterial strains and demonstrated antibacterial and antifungal effectiveness against *S. aureus* and *E. coli* (Figure 9) [52].

A novel gelatin nanocomposite hydrogel formulation encapsulated thyme essential oil in sodium caseinate nanomicelles formulated as a gelatin nanocomposite hydrogel which has been investigated as a drug delivery platform for in vitro antibacterial and in vivo wound healing potential [35]. The evaluation tests of the biocompatible and hemocompatible hydrogel showed a sustained in vitro release profile of EOs, with a strong antibacterial effect. In addition, the wound-healing potential of the nanocomposite was investigated in vivo, demonstrating a significant wound reduction in the group of animals it was tested on, after only 18 days. Antibacterial hydrogel may be a promising active and biocompatible platform for sustained delivery of thyme essential oil [35].

*Origanum vulgare* L. (oregano) has been used since ancient times all over the world, as a culinary ingredient, spice, or preservative and in curative treatments, being carminative, tonic, stimulant, and diaphoretic. Numerous studies have reported the main characteristics of this common plant, which demonstrate good analgesic, antimicrobial, antifungal, antiviral, antioxidant, and anti-inflammatory activities. In addition, it helps to easily penetrate the skin for transdermal drug administration [53]. In local applications, it is useful in antiaging treatments, due to its antioxidant and anti-inflammatory properties which provides protection against free radicals of various reactive oxygen species [54,55]. It has a wealth of volatile and non-volatile components such as flavonoids, phenolic acids, and tannins, mainly phenolic monoterpenes such as carvacrol and thymol, with a variable chemical profile depending on the species and the geographical area [56].

**Figure 10:**
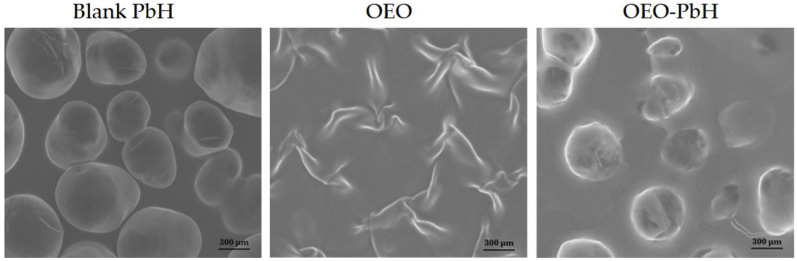
SEM micrographs of polymeric hydrogel (control), oregano essential oil, and micelle-based hydrogel sample containing oregano EO [57].

Recent research used oregano EO in an innovative hydrogel formulation based on polymeric micelles (Figure 10) [57]. The release and permeation profile of the EO, the in vivo effects on biocompatibility, and the impact of the hydrogel on in ovo-angiogenesis were evaluated. It should be noted that the study avoided animal testing and a chick chorioallantoic membrane was used. The results showed a sustained release of EO, having a potential anti-angiogenic effect. This hydrogel with oregano EO content could be a natural therapeutic alternative in skin pathologies, such as fibroepithelial polyps [57].

The bioactive and curative phytotherapeutic potential of essential oils has been exploited in a multitude of applications for wound healing [58,59].

Peppermint (*Mentha* × *piperita*) EO is widely used in the cosmetic industry for its aromatic fragrance. Its main constituents are menthol, menthone, and menthol acetate, and as secondary components, it contains bitter substances, caffeic acid, flavonoids, tannins, 1,8-eucalyptol, and propanone [60]. The pine needles EO mainly contains α-terpineol, linalool, and limonene, but also anethole, caryophyllene, and eugenol [61]. Fennel EO predominantly contains (E)-anethole, but also α-phellandrene and fenchone, methyl chavicol, p-cymene, and β-phellandrene [62].

**Figure 11:**
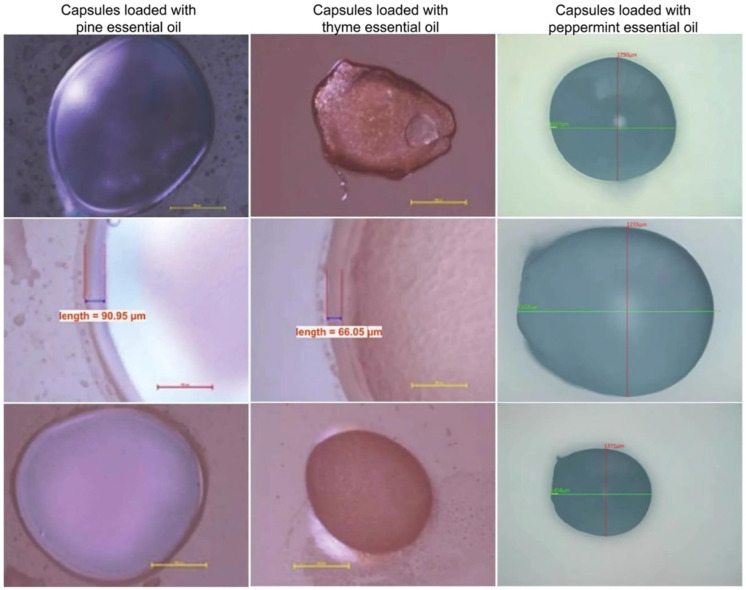
Optical microscopy images of polymer capsules of different shapes loaded with pine, thyme, and mint EO [63].

The healing capacities of four types of EOs have been used advantageously by encapsulating them in microcapsules in the first step, and then by incorporating them in polymer matrices in the form of films to develop dressings for wounds [63]. Polyvinyl alcohol, polyvinyl pyrrolidone, and hydroxypropyl methylcellulose were selected as polymeric materials. Poly(ethylene glycol) and glycerol were used as plasticizers, together with Zn stearate as a stabilizer, and vitamins A and E for the antioxidant effect. EOs of mint, thyme, pine, and fennel were loaded into the polymer matrices as active substances with antimicrobial effects. The different types and compositions of EOs and polymer components affect the shape and aspect of the microcapsules, which can be visibly observed (Figure 11). The results of the investigations showed that the samples made with EO content presented good inhibitory activity and antimicrobial properties against *Staphylococcus aureus*, *Enterococcus faecalis*, *Escherichia coli*, *Pseudomonas aeruginosa*, and *Candida albicans* [63].

#### 5.1.4. Chemotherapeutic

The alarming increase in the number of cancer cases in recent years highlights the pressing need for intensified efforts to improve therapeutic protocols [64,65]. Breast cancer, brain cancer, or invasive skin cancer affect millions of people every year and cause suffering and death all over the world [66,67]. With the multitude of different cancer treatment protocols, both surgical interventions and targeted therapies (chemo-, radio-, hormone-), the increase in the survival of cancer patients has been almost constant in recent years [68]. From here it is obvious the major urgency with which new therapies, combinatory, and targeted strategies are approached for a synergistic effect that will prolong the survival time and decrease the mortality rate [69,70].

Owing to some serious side effects of currently used anticancer chemotherapeutic methods or agents, there is a growing trend to use herbal medicine and its phytocompound derivatives [71]. It is important to use them both as ideal therapeutic alternatives and alongside chemotherapy treatments for many types of cancer [72,73,74].

Research is in a continuous dynamic and is actively focused on the discovery of new “green” pharmacological components for chemotherapies that offer potent potential activity with minimal side effects. A study aimed at obtaining new synergistic therapeutic agents (antimicrobial, antioxidant, and anticancer) was carried out by nanoencapsulation of clove essential oil in a nanogel based on squid chitosan and another phytochemical component, namely ρ-coumaric acid [75]. The in vitro evaluation of the nanogel encapsulated with clove essential oil indicated chemotherapeutic effects and potential for the prevention or therapy of pathologies induced by oxidative stress, microbial infection, or breast and skin cancer [75].

#### 5.1.5. Carrier for Drug Delivery

Hydrogel delivery systems are excellent therapeutic tools for multiple clinical uses [76]. The adjustable 3D structure of hydrogels allows the inclusion of small molecules, macromolecules, or growth factors and they have the ability to protect drugs susceptible to degradation. It also ensures precise spatial and temporal control over the release of therapeutic factors and degradability [77].

Bioactive molecule delivery systems are designed and developed in the form of films, pearls, and nanogels. In order to create a smart drug carrier with intestinal release activity, alginate hydrogel beads containing essential oils were made [78]. Glycyrrhizic acid, licorice root extract, and Thymus EO were loaded into ß-cyclodextrin. By co-encapsulating them with alginate, active alginate hydrogel beads were obtained. Studying the release of EO from alginate beads in simulated gastric fluid and simulated intestinal fluids indicated a high release rate of both EOs [78].

**Figure 12:**
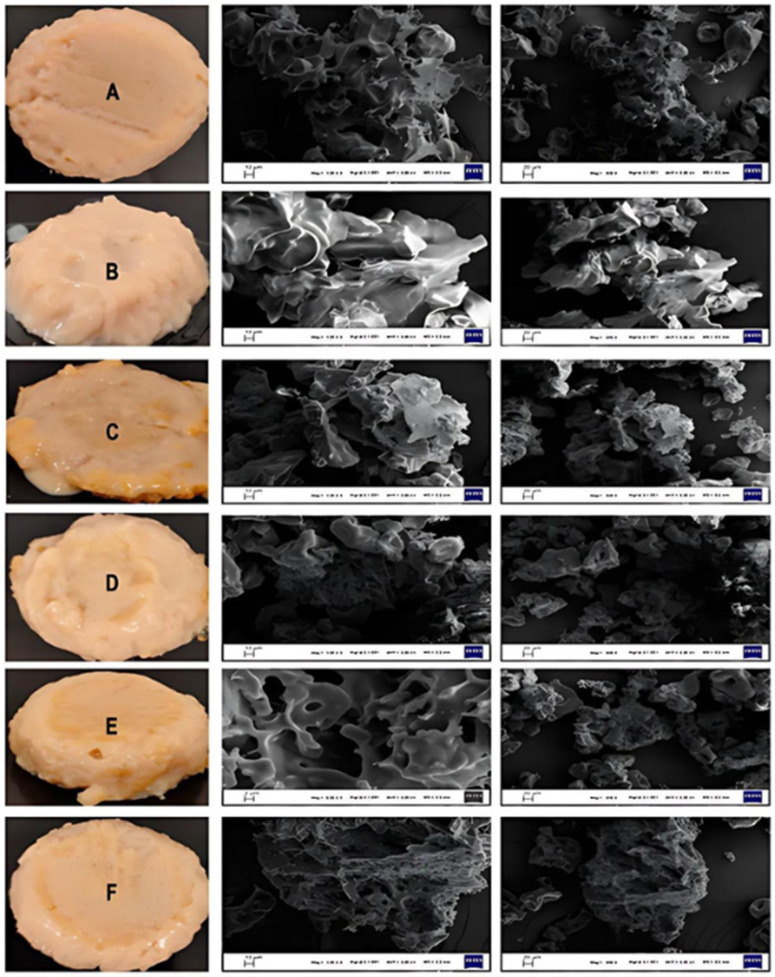
Photo and SEM images of different hydrogel formulations, with and without EO, nanoliposomes, and nanoliposome/maltodextrin complexes. (**A)**: control hydrogel; (**B**): hydrogel with EO; (**C**): hydrogel with lecithin and EO (14.23%); (**D**): hydrogel with lecithin, maltodextrin and EO (20%); (**E**): hydrogel with lecithin, maltodextrin and EO (25%); (**F**): hydrogel with lecithin, maltodextrin, and EO (33%) [79].

The characteristics of a natural hydrogel nanoliposome hybrid system were evaluated for the controllable release of thyme essential oil in the gastrointestinal tract [79]. Hydrogels based on pea protein and gum Arabic indicated the need for intermediates such as maltodextrin for stabilization. Figure 12 shows different photo and SEM images of different formulations, with and without nanoliposomes and nanoliposome–maltodextrin complexes [79].

#### 5.1.6. Burn Healing

Burns or scalds to the skin are particularly serious injuries, sometimes life-threatening, as they can disrupt the body’s essential functions. This disruption is primarily due to the loss of water, electrolytes, and proteins, because of the wounds [80].

Burns need emergency medical care along with very strict infection control and surveillance measures to increase the rate of healing and survival. The appearance of multi-resistant organisms to antibiotics or some treatments, such as dressings or ointments inappropriate for the degree of burn, can lead to invasive infections [81].

Polysaccharide-based hydrogel dressings are more advantageous materials for the treatment of burns, compared to traditional textile dressings, due to the easy application and removal and rapid coverage of the wounds together with the surrounding areas, the good capacity to absorb exudate, and the comfort given by the improvement quickness of pain. In addition, the transparency of most hydrogels allows for easier management of lesions.

Numerous studies have reported the results of the use of polymeric hydrogels with different EOs content in the care of wounds and burns [82,83,84]. New formulations of hydrogel materials based on polyvinyl alcohol and gelatin enriched with ginger extract have been proposed as dressings for burn wound healing. The hydrogels demonstrated comparable wound healing efficacy to the commercial dressing on rabbit back burn wounds in vivo. In addition, they showed significantly higher wound healing activity than the control group, as evidenced by intensive collagen development observed in histopathological analysis [85]. A new study obtained and tested materials for the treatment of burns by designing dressings based on physically cross-linked carboxymethyl chitosan and carbomer 940 hydrogels. EOs of eucalyptus, ginger, and cumin were selected and loaded into them [86]. The hydrogel containing eucalyptus EO showed favorable antibacterial activities against *S. aureus* and *E. coli*. Moreover, experiments performed in vivo on mice demonstrated that hydrogel with eucalyptus EO improved wound healing in burn models and considerably promoted the regeneration of the dermis and epidermis. The histological analysis highlighted the decrease in the values of IL-6, TNF-α, and the increase in the values of the factors TGF-β, VEGF, and EGF, specific to the burn wound tissue area [86].

### 5.2. Dental Applications

Traditionally, plants, herbal extracts, and essential oils have been successfully used in dentistry to clean teeth and dental caries [87]. People traditionally crafted toothbrushes using natural bristles from twigs selected from medicinal plants, which were rich in oils. Fir, clove, bay, eucalyptus, juniper, neem, or oak were used, with a rich content of volatile oils that acted to stimulate blood circulation, and with tannins for contraction and cleaning of the gums [88]. They also used poppies or cranberries, rich in vitamins, to keep their gums healthy. It has been observed that aloe vera plants, marigolds, and grapefruit seeds have beneficial and anti-inflammatory effects in the oral cavity [89]. They inhibit the growth of aerobic or anaerobic bacteria and act to reduce gingival bleeding and gingivitis [90].

Phytochemicals provide a potential strategy in the prevention and treatment of dental caries, inflammation, and other oral infections and could be a powerful substitute for antibiotics [91,92].

A promising strategy for the prevention and treatment of dental caries, inflammation, and other oral infections is the use of phytochemicals both in current care products and in oral treatments [87]. These natural compounds could serve as a powerful alternative to antibiotics [93].

Extensive recent research has developed hydrogels with incorporated essential oils for the therapy of periodontitis [94]. These materials are described as dental drugs that could be used as photosensitizers in photodynamic therapy for the treatment of periodontitis. Oregano^®^, Frankincense^®^, and the Thieves^®^ blend were incorporated as EOs, with a content of cloves, lemon, cinnamon bark, eucalyptus radiata, and rosemary extract. The main constituents identified from the mixture of selected and used oils included eugenol, pinene, limonene, carvacrol, and cymene [94].

### 5.3. Cosmetics Applications

Essential oils are integral to the formulation of care products and cosmetics, offering a wide range of benefits thanks to their rich and diverse composition of biocompounds [95,96]. Moreover, hydrogels combined with various chemical compounds can be incorporated into cosmetic formulations, offering multiple topical applications for both skin and hair [97,98].

Hydrogels for cosmetic preparations can be obtained from biopolymers of natural origin, such as alginate, collagen, gelatin, hyaluronic acid, chitosan, xanthan gum, pectin, starch, or cellulose [99,100]. These biopolymers themselves possess bioactivities advantageous to cosmetics. Thus, new cosmetic products were designed and made in the form of gels, microcapsules, or masks, both for skin and hair, with excellent hydration, softening, and elasticity performances, supporting and actively promoting anti-aging. Also, superabsorbent hydrogels have been developed in comfortable hygiene products, capable of absorbing fluids.

The combination of hydrogels with essential oils is a successful mixture, particularly useful and advantageous as cosmetic preparations or beauty and care products [101,102,103].

Recently, a study was reported that aimed at the design and creation of new cosmeceutical materials based on hydrogels with improved biological properties [104]. In the first step, *Camellia oleifera* EO was loaded into chitosan nanoparticles by emulsification and then ionic gelation. Then, hydrogels based on poly(vinyl alcohol), silk sericin, and gelatin were prepared, in which chitosan nanoparticles were embedded. Materials that showed tyrosinase inhibition and antioxidant activity could be useful in cosmeceutical applications, such as facial masks [104].

### 5.4. Food Applications

Hydrogel-based formulations with incorporated EOs have numerous applications in the food industry [105].

Hydrogels for food application should be categorized as follows: (i) Delivery; (ii) Packaging; (iii) Coating; (iv) Fat replacer; and (v) Texturizing. A growing field of research centers on hydrogel beads, which act as carriers for nano- or microparticles. These systems are highly effective for targeted drug delivery and can also be used as food supplements, including dietary additives, probiotics, or food components for special medical purposes [105,106]. These types of materials, which can be administered orally, have exploited the biological origin of natural polymers, especially polysaccharides and proteins, their specific biodegradability, and pH sensitivity [107]. A considerable amount of research has been devoted to the development of hydrogels for the encapsulation of food-grade components, such as vitamins, natural extracts, and essential oils [108].

Hydrogel beads show great potential for improving the bioavailability and performance of some compounds from the range of nutraceuticals, including EO, offering them protection against chemical degradation [109]. The granules are made through an accessible technique, in two steps: (i) obtaining the particles enriched with biopolymeric materials and nutraceutical content, and (ii) crosslinking the biopolymeric materials. The first step can be achieved by injection, phase separation, shearing, or templating, and the second step can be achieved by degree changes in the solvent quality, the incorporation of counter-ions or enzymes, or by heating–cooling cycles [110].

Recently, substantial amounts of waste from coffee pulp, generated during the extraction of essential oils, were analyzed [111]. These wastes were used to extract two different pectin fractions (highly methoxylated and low methoxylated). Pectins have been studied for their performance as EO carrier systems. The pectin fractions formed two systems of hydrogel beads, with or without chitosan, to encapsulate the EO of roasted coffee or green coffee. The two systems were analyzed in terms of their antioxidant activity and EO release profile for potential food applications. On the one hand, the highly methoxylated pectin obtained from *Coffea arabica* presented better EO encapsulation performances. On the other hand, surprisingly, the EOs obtained from roasted coffee showed superior antioxidant activity compared to that obtained from green coffee [111].

Food packaging serves as a passive barrier, shielding products from environmental factors, extending their shelf life by preventing contamination, and ensuring safe transportation and storage. Active packaging incorporated with essential oils allows interaction between food and the external environment, helping to regulate temperature, moisture levels, and microbial control, which ultimately enhances the quality and extends the shelf life of the food [112]. The upcoming chapter will focus on the key concerns and challenges faced by the food packaging industry. It will explore how essential oils influence the microstructure of packaging materials and examine their specific properties.

A new direction regarding the applications of hydrogels with EO content is represented by edible coatings that extend the shelf life of some perishable foods, by delaying oxidation and reducing the amount of packaging. EOs are known to be excellent natural antimicrobial and antibacterial agents [113]. Some contaminants such as gram-negative bacteria (*E. coli*) can cause serious diseases by contaminating food such as milk and meat or gram-positive ones (*S. aureus* and *B. cereus*), which cause the contamination of fruits or food products with starch content.

The richness of volatile compounds contained in EO, such as phenolics, determines the use of oils in edible films or coatings for flavoring, packaging, or preservation of food products. Different gels incorporating EO such as basil leaves, clove, cypress, fennel, lavender, oregano, pine, rosemary, thyme, and verbena have been used to inhibit lipid oxidation and microorganism growth in coatings for fish fillets, cheese, fruits, or vegetables [114,115,116,117,118].

Studies on two types of gelatin hydrogels containing rosemary and orange EO microdroplets prepared by simple emulsification in the presence of Tween^®^80 surfactant showed interesting conclusions [119]. The mechanical and antibacterial properties of these gels against some food contaminants such as *E. coli*, *S. aureus*, and *B. cereus*. indicated adequate characteristics as edible coatings of perishable foods, in order to preserve foods such as meat [119].

A novel area of research that has gained attention recently is the replacement of animal fats through the immobilization of oils within hydrogels [120]. Healthier meat products are a direction imposed both by worldwide recommendations and by consumer demands. Traditional products should be adapted to the nutritional characteristics recommended by specialists, by reformulating them. Therefore, a recent approach to improving the health of meat products is the use of healthy oils (vegetable or marine) as fat substitutes. Also, it is important to develop food products that are low in fat, but which retain their functional qualities, such as mayonnaise or ice cream [121]. However, it is a great challenge for researchers to keep the specific bioactivities of hydrogels with incorporated essential oils and use them as fat substitutes or as materials with specific textures. Future scientific discoveries based on nanotechnology will also develop such products.

### 5.5. Food Packaging Applications

Active packaging is representing intelligent materials that improve the preservation of food, especially perishable ones, extend the shelf life, and ensure safety by interacting with the food product through its various components [122].

Currently, there is a multitude of advantageous active packaging for applications in different fields. In the food industry, but also in the beverage industry, there is the most active packaging, due to the very high demand for increased shelf life, freshness, and safety. The pharmaceutical industry, medical technology, agriculture, and courier and delivery services are just a few other areas where there is a demand for these types of modern packaging.

Active packaging is created based on the active components of biopolymers or with different biocompounds incorporated into them [123].

For instance, biopolymeric hydrogels with essential oils incorporated as antimicrobial substances are advantageous systems for obtaining active food packaging. In addition to monitoring the condition and ensuring food safety, minimizing the risk of contamination, increasing the shelf life, or obtaining more durable packaging, smart packaging can help reduce food waste. In this context, adding EOs to packaging is a natural alternative that can replace chemical additives [124,125].

Cinnamon EO is a natural bacteriostatic agent, with potential applications in the field of food preservation [126]. In general, it has found many uses in culinary and medicinal applications. It contains numerous chemical constituents, of which, depending on the different species of *Cinnamomum* trees or shrubs, the most important are the compounds (E)-cinnamaldehyde, linalool, β-caryophyllene, eucalyptol, and eugenol [126]. Apart from the specific spicy taste and cinnamon flavor that is due to the cinnamaldehyde compound, the wide variety of components of cinnamon EO have antimicrobial, antioxidant, antifungal, and antidiabetic biological properties [127,128].

**Figure 13:**
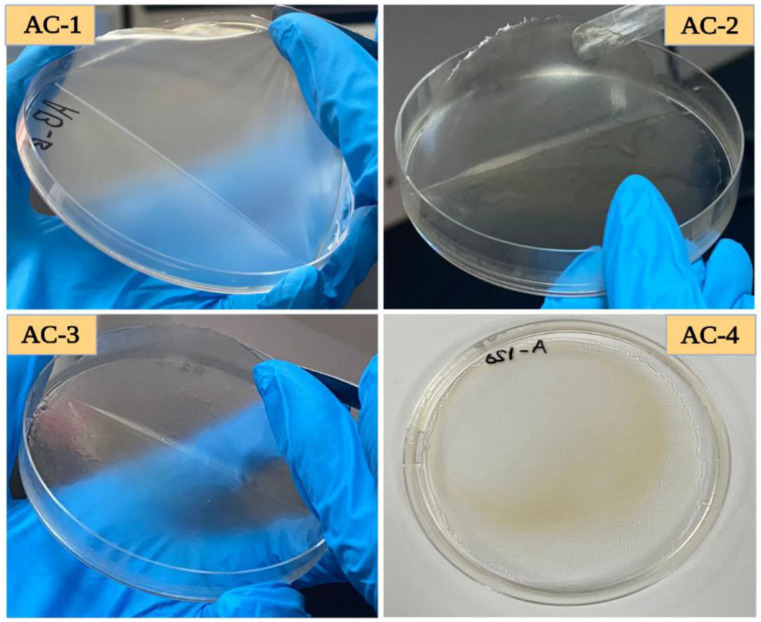
Photo of the composite films based on sodium alginate and acacia gum with different concentrations of cinnamon EO: (AC1—without EO) (AC2—15 μL EO) (AC3—20 μL EO) (AC4—30 μL EO) [129].

Sodium alginate and acacia gum hydrogels loaded with cinnamon EO were prepared as edible films and analyzed in terms of view of physicochemical characteristics [129]. The antioxidant capacity of the films was improved with increasing cinnamon EO concentration, making them promising candidates for use as active food packaging materials. Figure 13 shows the composite films prepared with different concentrations of cinnamon EO between 0–30 μL EO [129].

Recently, the phytochemical components of the essential oil obtained from the *Artemisia dracunculus* plant, widely distributed geographically, were identified and evaluated [130]. *Artemisia dracunculus* EO was valorized by incorporating different amounts into hydrogel matrices based on polyvinyl alcohol and agar. The results of the antimicrobial tests indicated sustained antimicrobial activity against nine pathogenic strains (four Gram-positive and five Gram-negative). The incorporation of *Artemisa dracunculus* EO in these hydrogel models can lead to practical applications in the area of food technology, as an active and biodegradable alternative to classic packaging [130].

In another study, a quantity of powdered starch was obtained in the first stage from the use of residual biomass, then in the second stage, it was introduced into a formulation, to prepare cryogels and hydrogels [131]. The materials prepared by absorption or cross-linking by the Schiff-base reaction were loaded with diacetyl and mint EO. The prepared materials showed a good ability to adsorb water and deliver antimicrobial substances, being advantageous for possible fresh food packaging applications [131].

Furthermore, new antibacterial hydrogels were prepared by the method of freeze–thaw cycles [127]. Inclusion complexes methyl-β-cyclodextrin and thyme oil were incorporated into a polyvinyl alcohol matrix with polysaccharide content, respectively, dendrobium and guar gum, in various ratios. These materials presented very good mechanical performance, as well as antimicrobial and antioxidant activities favorable for the preservation of chicken breast, extending the shelf life by four days. These results indicate the potential of the materials for possible active packaging applications [132].

### 5.6. Restoration of Stone Cultural Heritage

Stone monuments in the sphere of cultural heritage suffer from biological damage. The variation in environmental conditions determines the growth of phototrophic microorganisms on stone surfaces, in the form of biofilms [133,134,135]. These microorganisms are made of microbial aggregates that act mechanically and produce micro-decohesion of the substrates [136]. In addition, biofilms, together with atmospheric pollutants, promote chemical corrosion, pigmentation, or discoloration of stone surfaces [137].

The classic restoration of stone surfaces uses both physical and chemical methods [138]. However, mechanical brushing can damage the surface of the monument, and chemical treatment can lead to a selection of resistant microbial species or can be harmful to the environment or the operators [139,140].

An innovative and eco-sustainable restoration technique is the use of essential oils with natural biocidal action, embedded in hydrogels, as alternatives to chemical treatments for the restoration of cultural heritage [141,142,143,144].

EOs of lavender and thyme were encapsulated in alginate hydrogel in order to create an easy-to-use and non-invasive restoration method [136]. The vitality of cyanobacterial biofilms was discouraged by applying hydrogel for different periods of time. The results of the tests indicated that the best inhibitory effect on the photosynthetic activity of microorganisms was shown by thyme oil rich in thymol, for a concentration of 0.1% (*v*/*v*) it was shown by thyme oil rich in thymol. It retained an effective antimicrobial action against cyanobacteria. Notably, the developed protocol allowed the use of a very small amount of essential oil as a green biocide [136].

In another study, thyme EO was also used in the preparation of poly(vinyl)alcohol and borax-based hydrogels, together with a double-layered hydroxide of ZnAl intercalated with sodium alginate, and silver nanoparticles or a mix of silver–silver chloride nanoparticles [145]. The hydrogels were thus formulated to mechanically remove the biopatina from two types of biodamaged stones: Carrara marble and St. Margarethen. The hydrogel with thyme EO content worked effectively for cleaning stones with porous structures and different compositions, damaged by the natural environment [145].

A real case study reported the results of in situ application of a sodium alginate hydrogel containing thyme EO. The experiment followed the restoration of three selected parts of Fortunato Depero’s mosaic located in a neighborhood in Rome (Italy) [146]. The material was prepared by a simple method and easily applied on large and vertical surfaces. The images taken before and after application demonstrated that a single treatment was enough to completely eliminate the microbial patina. The hydrogel loaded with thyme EO as a natural biocide showed a very good biocide performance [146].

In summary, Table 1 outlines the key characteristics of various essential oils discussed in this review, highlighting the main bioactive compounds, the extraction methods used, pharmacological features and potential applications. The brief summary of essential oil-enriched hydrogel applications introduced in this manuscript can be found in Table 2.

## 6. Challenges and Perspectives

The advancement of polymeric hydrogels presents emerging opportunities for their use across various fields, thanks to their biocompatibility, simple gelation process, ease of application, and the potential for functionalization. Hydrogels have the ability to alter their volume, phase, and structure when exposed to specific external stimuli, making them versatile for use in a wide range of sectors. However, the limited mechanical rigidity commonly found in some biocompatible hydrogels presents a significant challenge that needs to be addressed, particularly when dealing with rapid dynamic changes or when considering structural uniformity and long-term stability.

Integrating EO into the hydrogel matrix can enhance their biological activities, shield them from degradation, and serve as a platform for creating innovative biotechnological products. Furthermore, encapsulation seeks to address certain limitations of EOs, such as their volatility, reduced stability, and high sensitivity to environmental conditions. The controlled release of bioactive compounds from EOs encapsulated within hydrogels is crucial for effectively delivering these compounds to their target. On the other hand, a huge difficulty, especially in medical applications, is the very high qualitative and quantitative variability of the EO composition. This is determined by intrinsic factors that influence each other and are due to the varieties and age of the plants, the type of soil, the climate, or the time of harvesting, but also by some extrinsic factors such as the extraction methods used.

However, to enhance their effectiveness as targeted delivery systems of EOs, further research is required to assess their safety across various applications, ranging from biomedical to food industries.

The limitations related to the insufficient data on the stability, safety, and long-term bioactivity of these materials are emphasized. Moreover, the limited number of in vivo studies, particularly in the medical field, could delay their commercialization in the pharmaceutical and biomedical sectors.

## 7. Conclusions

This review aims to provide an overview of polymeric hydrogels containing essential oils, emphasizing their vast potential for applications in various fields. Hydrogels are valuable as delivery systems because of their ability to be biocompatible, biodegradable, and provide controlled release of plant-derived bioactive ingredients. Hydrogel structures, known for their remarkable swelling, gelling, and bioactive loading capabilities, play a vital role in the creation of functional materials. Most essential oils are accepted, credited, and appreciated as valuable bioactive ingredients, capable of performing multiple pharmacological functions, such as anticancer, antiseptic, antiviral, and antioxidant activities. Results from the cited literature suggest that hydrogels containing essential oils are ecological, sustainable materials, with improved biological properties, demonstrating effective antibacterial, antifungal, anticancer, and anti-inflammatory activities. Nevertheless, challenges remain in this field, including the need for standardization and the absence of cost-effective methods for scaling up production on a larger scale. Additionally, issues concerning stability and toxicity require thorough investigation. The advancement of essential oil-enriched hydrogel materials presents a growing opportunity to be applied in a wide range of fields, including biomedicine, cosmetics, dentistry, the food industry, and even heritage conservation.

## Figures and Tables

**Table 1:** General characteristics of essential oils incorporated in hydrogels.

Plants	Essential Oils	Main Constituents	Extraction Procedure	Pharmacological Properties	Applications	Reference
*Cinnamomum zeylanicum*	Cinnamon oil	cinnamaldehyde	Steam distillation and Soxhlex extraction	Antimicrobial, antibiotic, antioxidant	Food packaging materials, food preservation	[129,147,148,149]
*Lavandula angustifolia*	Lavandin essential oils	Terpenes (e.g., linalool, linalyl acetate, terpinen-4-ol) and terpenoids (e.g., eucalyptol)	Steam distillation	Antioxidants, antibacterial, anxiolytics, analgesics, and anti-inflammatories	Wound healing, Microparticles as delivery system	[48,150,151,152,153]
*Cymbopogon* (spp.)	Lemongrass essential oils	Terpenes and Terpenoids (Terpinen-4-ol, α-Terpineol (neral, isoneral, geranial, isogeranial, geraniol, geranyl acetate, citronellal, citronellol, germacrene-D, and elemol)	Steam distillation	Antifungal, antibacterial, antiviral, anticancer, and antioxidant	Pharmaceutical, cosmetics, and food preservations industries	[154,155]
*Melaleuca alternifolia*	Tea tree essential oils	Terpenes (e.g., terpinen-4-ol, 1,8-cineole)	Steam distillation	Antimicrobial and anti-inflammatory	Beads for food preservation	[156,157,158]
*Mentha piperita*	Peppermint essential oils	Menthol, menthone, neomenthol and iso-menthone	Steam distillation, hydrodistillation, microwave-assisted extraction, supercritical fluid extraction, ultrasonic-assisted extraction and countercurrent extraction	Anti-inflammatory, antibacterial, antiviral, scolicidal, immunomodulatory, antitumor, neuroprotective, antifatigue and antioxidant; hypoglycemic and hypolipidemic effects, gastrointestinal and dermatological diseases	Patches, wound dressing	[63,159,160,161,162]
*Ocimum basilicum* (L.)	Basil essential oils	Eugenol, e α-Pinene, β-Pinene, Methyl chavicol, 1,8 cineole, L-linalool, Ocimene, Borneol, Geraneol, B-Caryphyllone, and n-Cinnamate	Hydrodistillation	Carminative, galactogogue, stomachic and antispasmodic tonic, vermifuge,	Food packaging, antiperspirant in agriculture	[163,164,165,166]
*Thymus vulgaris* (L.)	Thyme essential oils	Carvacrol, 5-isopropyl-2-methylphenol, and a p-cymene	Hydrodistillation, steam distillation	Antioxidant, antimicrobial, antidiabetic, anti-inflammatory, immunomodulatory and anticancer bioactivities	Wound healing, wound dressing; beads as delivery systems	[33,35,36,47,63,79,167]

**Table 2:** Applications of essential oils incorporated in hydrogels.

	Method of Preparations	Materials	Encapsulated Essential Oils	Applications	References
Biomedical applications	Physical crosslinking	Sodium alginate/Fucoidan	Menthol, L-linalool, bergamot oil, and β-pinene	Topical or transdermal administration	[43]
	Physical crosslinking	Methylcellulose (10% (*w*/*v*))	*Melissa officinalis* EO	Treatment of oral candidiasis.	[44]
	Chemical crosslinking	Polyvinyl Alcohol/Corn Starch Hydrogel Films loaded with Silver Nanoparticles	Patchouli EO	Antimicrobial materials (against *Staphylococcus aureus* and *Staphylococcus epidermidis*)	[45]
	Solvent displacement method	Poly-(D,L)-(lactic-co-glycolic acid)	Thyme EO	Inflammatory skin disorders	[47]
	Physical crosslinking	Polyvinyl alcohol/kaolin	Cedar EO	Wound dressing	[49]
	Covalent and physical crosslinking	Chitosan/oxidized pullulan	Clove EO	Wound dressings	[52]
	Cold gelation process	Polymeric-Micelles-Based Hydrogels (Pluronic F127–20%*w*/*w;* and Pluronic L 31—1%*w*/*w*)	Oregano EO	Cutaneous application	[57]
	Chemical crosslinking; casting method	Polyvinyl alcohol/polyvinyl pyrrolidone; hydroxypropyl methyl cellulose; sodium alginate; polyethylene glycol; glycerol; Zn stearate; vitamin A and E	Fennel, pine, mint and thyme EO	Wound dressings	[63]
	Chemical crosslinking	Ultrasound-assisted deacetylated chitosan/ρ-coumaric acid	Clove EO	Chemotherapeutic/chemopreventive agent	[75]
	Thin-film dispersion technique/heat-induced gelation.	Pea protein (30%) and gum Arabic (1.5%); Soybean lecithin; maltodextrin and gum Arabic	Thyme EO	Delivery of bioactive compounds (food packing material, tissue engineering or drug delivery)	[79]
	Physical crosslinking	Carboxymethyl chitosan/carbomer 940	Eucalyptus, ginger, and cumin EO	Burn dressing material for skin burn repair	[86]
Dental applications	Physical crosslinking	Xanthan gum/Glycerin/Lyophilized Whey/Polyvinylpyrrolidone/PEG 400	Oregano^®^, Frankincense^®^, Thieves^®^, Frankincense^®^ EO	Therapy of periodontitis	[94]
Cosmetics applications	Ionic gelation	Poly(vinyl alcohol), silk sericin, and gelatin/chitosan nanoparticles	*Camellia oleifera* EO	Cosmetic product (facial masks)	[104]
Food applications	Emulsification/ionic crosslinking	Pectin and pectin/chitosan hydrogel beads	Green and roasted coffee EO	Systems for the delivery and controlled release of essential oils; food applications	[111]
Food packaging applications	Gelation/casting	Sodium alginate/acacia gum	Cinnamon EO	Hydrogel-based films as active food packaging materials	[129]
Restorations of the stone cultural heritage	Preparation directly in situ	Sodium alginate	Thyme EO	Biocides for restoration in a real case of study, i.e., the mosaic Le Professioni e le Arti of Fortunato Depero	[146]

## Data Availability

Not applicable.
